# Psychosocial and Diet-Related Lifestyle Clusters in Overweight and Obesity

**DOI:** 10.3390/ijerph18126461

**Published:** 2021-06-15

**Authors:** Débora Godoy-Izquierdo, Raquel Lara, Adelaida Ogallar, Alejandra Rodríguez-Tadeo, María J. Ramírez, Estefanía Navarrón, Félix Arbinaga

**Affiliations:** 1 Departamento de Personalidad, Evaluación y Tratamiento Psicológico, Facultad de Psicología, Campus Universitario de Cartuja, Universidad de Granada, 18071 Granada, Spain; adelaidaogallar@ugr.es; 2 Grupo de Investigación Psicología de la Salud y Medicina Conductual (CTS-267), Centro de Investigación Mente, Cerebro y Comportamiento, Facultad de Psicología, Campus Universitario de Cartuja, Universidad de Granada, 18071 Granada, Spain; rlaramoreno@ugr.es (R.L.); mariaps@correo.ugr.es (M.J.R.); enava@correo.ugr.es (E.N.); 3 Departamento de Psicología Social, Facultad de Psicología, Campus Universitario de Cartuja, Universidad de Granada, 18071 Granada, Spain; 4 Departamento Ciencias de la Salud, Instituto de Ciencias Biomédicas, Anillo Envolvente del Pronaf y Estocolmo, Universidad Autónoma Ciudad Juárez, Ciudad Juárez 32300, Chihuahua, Mexico; alrodrig@uacj.mx; 5 Departamento de Psicología Clínica y Experimental, Facultad de Educación, Psicología y Ciencias del Deporte, Campus Universitario El Carmen, Universidad de Huelva, 21071 Huelva, Spain; felix.arbinaga@dpsi.uhu.es

**Keywords:** body image, healthy diet, weight-related stigma, subjective well-being, excessive weight, cluster analysis

## Abstract

This study explored intraindividual multidimensional profiles integrating psychosocial factors, namely, body image and satisfaction, weight-related self-stigma, positivity, and happiness, and behavioural-lifestyle factors, namely, adherence to a healthy diet, among Spanish adults with overweight or obesity. We further aimed to investigate the association of excess weight (i.e., measured body mass index, BMI) with the abovementioned multidimensional configurations. A convenience sample of 100 adult individuals (60% females) with excessive weight (69% overweight; 31% obesity) was recruited. They completed self-reports regarding the study variables, and their weight and height were measured. With a perspective centered on the individual, a cluster analysis was performed. Three distinct intraindividual psychosocial and diet-related profiles were identified: a group of healthy individuals with excess weight (46%); a group of individuals who were negatively affected by their excessive weight and showed the most distressed profile (18%); and a group of dysfunctional individuals who seemed to be excessively unrealistic and optimistic regarding their excessive weight and unhealthy lifestyles, but were troubled by their weight (36%). Furthermore, individuals in the affected cluster had higher obesity (mean BMI ± *SD* = 32.1 ± 3.7) than those in the clusters of healthy (28.0 ± 3.0) and dysfunctional individuals (28.1 ± 3.3) (*p* < 0.05). The results showed that there are specific psychosocial and lifestyle profiles in the adult population with excess weight and that there are relationships among psychological, behavioural, and body-composition factors. For clinical application purposes, it is important to account for the heterogeneity within individuals who are obese and to individualize the interventions, with a focus from weight change to the individual’s overall well-being.

## 1. Introduction

Overweight (body mass index, BMI ≥ 25 kg/m^2^) and obesity (BMI ≥ 30 kg/m^2^) are recognized as major public health concerns, as they are associated with a higher risk for chronic or severe somatic, mental, and social co-morbidities. Individuals with overweight and obesity form a heterogeneous population in terms of etiological factors, the ways in which being obese impacts an individual’s health and functioning, how the individual sees himself or herself, and how he or she is seen by the world [1]. Consequently, they have different needs. Thus, a better conceptualization of subgroups among overweight/obese individuals will lead to more specific and tailored interventions and body weight management strategies [2].

Obesity is categorized based on weight/BMI and related morbid conditions; however, such a distinction fails to account for the variation within this group across other factors, such as demographic factors, health-related variables, and psychosocial and behavioural characteristics [3]. Despite the multifactorial nature of obesity and the heterogeneity of weight-related experiences, there has been little work categorizing multidimensional patterns, which might allow for the tailoring of interventions. Contrary to considering obesity as a single discrete factor, in isolation from other factors [3], the exploration of how a variety of demographic, psychosocial, lifestyle, health-related, and well-being factors are interrelated among and between groups of individuals will allow for the understanding of the diversity of obese individuals and the derivation of customized interventions. Analytical procedures for clustering individuals based on their commonalities provide a novel integrated investigation approach and offer guidance for intervention designing, tailoring, and targeting [4].

After initial and more recent research focused on subtyping overweight and obese individuals based on psychopathological risk factors for obesity [5,6,7,8], the efforts for empirically deriving “typologies” of experiences with excessive weight have more recently been focused on distinguishing between more adaptive profiles and less positive other ones. A range of modifiable obesity-related factors has been considered, including identity statuses [1,9], weight-gain behaviours [3,4], body image concerns [1,10], life satisfaction [3], negative affect [2,10], and eating pathology [2,11]. Overall, this research supports the heterogeneity of individuals with excess weight, and two to three clusters have been found: one adaptive/high-functioning subgroup of individuals with excessive caloric intake and unhealthy diet and no evidence of relevant psychopathology including disturbed eating, and two maladaptive/distressed/undercontrolled subgroups of individuals with increased body dissatisfaction, unhealthy or disordered eating, and general psychopathology (e.g., depression), where unhealthier patterns of body concerns and eating-related disturbances are associated with poorer mental health. These profiles were also different in terms of validation, external variables of psychosocial functioning, mental health, obesity-related quality of life, and eating disturbance. Nevertheless, this research has been restricted mainly to high-risk or morbid female samples or individuals undergoing weight treatment and has focused on a limited number of configuration variables. Research on multidimensional psychosocial profiles including a variety of behavioural-, health-, and well-being-related variables among a broad range of excessive BMIs is scarce.

Given the heterogeneity of individuals with excessive weight, the scarcity of research on multidimensional profiles in overweight and obesity, and the necessity of tailoring interventions to the features of each of the subgroups within this population, it is extremely necessary to investigate an empirically based categorization of individuals with excess weight based on several psychosocial, lifestyle, and health-related variables. Shape–weight concerns, weight-related stigma, and psychological distress have been found to define, in combination, a psychosocial profile of experiences with obesity among individuals with excessive weight [12]. In addition to psychopathological characteristics, unhealthy attitudes and behaviours, the risk of disease and eating disorders, weight loss failure, and diminished quality of life, research on the positive states of well-being and protective factors configuring alternative psychosocial profiles is unfortunately scarce [13]. Thus, an integration of several psychosocial and behavioural variables related to obesity, such as body image, social stigma, personality, lifestyle habits, and subjective well-being (SWB), can be helpful in identifying patterns of relationship between these factors and understanding subtypes of individuals with excessive weight.

Given that negative body image and body dissatisfaction [14,15,16,17,18,19,20,21,22] and weight-related prejudice, discrimination, and stigma [13,23,24,25,26,27] have been consistently found to be related to decreased functioning and well-being, these variables are worthy of being considered in a configurational analysis. 

In addition, whereas the extant research has focused mostly on the harmful consequences of obesity in terms of psychological ill-being, it is also interesting to explore positive health and well-being indicators in individuals with excessive weight, such as happiness [3,28,29,30,31,32,33,34,35,36,37,38,39]. To the best of our knowledge, protective personality resources have not been previously incorporated into the configuration of psychosocial profiles among individuals with excess weight. A valuable healthy personality trait is positivity, a positive orientation towards self, life, and the future [40,41], which has not been extensively investigated up to now in obesity [13]. 

Besides, life-style factors (e.g., over- and malnutrition, sedentary lifestyle) should be considered. A healthy and diverse eating pattern is a widely accepted recommendation for promoting a nutritionally adequate diet and general health and for reducing the risk of major chronic diseases, including obesity [42]. The Mediterranean diet (MedDiet) is characterized, among other features, by an adequate intake of plant foods, such as fruits and vegetables (FV), which are associated with a healthy body weight and the prevention and management of obesity and other chronic and fatal diseases, including cardiovascular disease and cancer [42,43,44,45,46,47,48,49,50]. The benefits of FV intake on health endpoints seem to be associated with an optimal daily intake ≥ five portions/servings [43,48,51]. Although Spain is considered to follow a MedDiet eating pattern, the reality is that the adherence to recommendations among Spanish individuals is far from ideal, and there is low FV consumption, which is also associated with the increasing national rates of overweight and obesity [51,52,53,54,55]. Recent studies suggested that the percentage of individuals who report never consuming or consuming <5 servings per day of FV is significantly higher in those with obesity than in those with normal weight [30,51,56]. Thus, an indicator of healthy diet such as FV intake is worthy to be considered as well in a multidimensional configurating analysis.

In the present study, we aimed to explore possible configurations of psychosocial and lifestyle factors, i.e., body image, weight stigma, positivity, healthy diet, and happiness, among Spanish adults with overweight and obesity. Together, these factors have not been explored to date. We further aim to validate such profiles by exploring possible differences due to BMI levels. Given that we intend to discriminate individuals in terms of specific configurations of a wide range of factors that are associated to healthier and unhealthier experiences of excessive weight, exploring whether such profiles are different based on BMI status is a form of validating the obtained profiles.

Based on previous research [1,2,4,6,8,11], we expected to find three different profiles, including a subgroup of individuals with more positive body perceptions, higher body satisfaction, lower self-stigma, healthier diet, positive orientation, and higher levels of happiness, and two distinct profiles of maladaptive combinations of such psychosocial and lifestyle factors, one revealing higher body- and social-related concerns (i.e., characterized by elevated body dissatisfaction, higher self-stigma, and healthier diet for weight control) and another revealing increased weight-related distress (i.e., characterized in addition by lower positivity and happiness). We also expected to find that the latter will also show the highest BMI.

## 2. Materials and Methods

### 2.1. Participants and Procedure

A total of 100 adults from 19 to 57 years old (average age: 42.03 ± 10.74 years, 60% women) residing in southern Spain voluntarily participated in the study. All participants had a BMI ≥ 25 (69% overweight: 45% overweight I and 24% overweight II; 31% obesity: 22% obesity I and 9% obesity II). No differences were found in terms of age between women and men or between overweight and obese participants. All participants were Mediterranean, white, and had an average socioeconomic status (social class was determined by combining education level, job status, and income; following the definition of the Health Determinants Taskforce of the Spanish Epidemiology Society, six categories were established and regrouped into three categories: high (Classes I-II), middle (Classes III-IV), and low (Classes V-VI) social classes [13]). 

Recruitment was conducted through a convenient, nonprobabilistic procedure according to the inclusion criteria (i.e., having overweight or obesity, not suffering from severe physical and mental diseases, and being 18–65 years old) in local medical settings. The sample size was estimated prior to the study using the Clinical and Translational Science Institute (University of California, San Francisco) online calculator for clinical correlational research [57] in 40 to 140 participants for alpha = 0.05, beta = 0.02, and expected rs for several associations among the study variables previously reported. We thus decided to recruit as many individuals as possible. The sample was finally composed of individuals with excess weight who sought consultation for weight and health in two collaborating primary health care centers during the approved period for recruiting participants and conducting the assessment phase (March 2019). Those who met the inclusion criteria and agreed to voluntarily participate collaborated. 

After inviting individuals to voluntarily participate and informing them about a study on well-being and health in obese and overweight adults, the anonymous nature of the data, and their rights, we obtained their written informed consent. Then, an assessment was conducted in a medical examination room. First, sociodemographic data and self-reported weight and height were collected in an interview format. Then, participants’ self-reported data on body perceptions, self-stigma, positivity, happiness, and adherence to the MedDiet were collected. The order of the questionnaires was counterbalanced to avoid order bias. Finally, objective measures of weight and height were obtained.

Approval was obtained from the ethics committee of authors’ university (CIEB-2018-1-36). The procedures used in this study adhere to the tenets of the Declaration of Helsinki of 1975–2013.

### 2.2. Study Variables and Measures

Sociodemographic data were collected from participants. We measured weight and height with a mobile anthropometer (Aicok Weight Scale, mod. CF398BLE, Beijing, China), which uses bioelectrical impedance analysis technology to monitor multiple physical indexes, including body weight (weight range up to 400 pounds/180 kg and indexing value accurate up to 0.2 pounds/0.1 kg) and BMI. Participants were weighed while erect, with their arms along their body, in bare feet and light clothes. BMI was then categorized according to international standards for nutritional status in the adult population [58,59]; i.e., <18.5 kg/m^2^ denotes low weight, 18.5–24.9 kg/m^2^ denotes normal weight, 25.0–29.9 kg/m^2^ denotes overweight (25.0–26.9 kg/m^2^ denotes overweight type I, and 27.0–29.9 kg/m^2^ type II), and ≥30.0 kg/m^2^ denotes obesity (30.0–34.9 kg/m^2^ denotes obesity type I, 35.0–39.9 kg/m^2^ type II, and ≥40.0 kg/m^2^ type III).

The perceptual component of body image [60] was explored by using silhouettes corresponding to different BMI ranges [16]. A total of 15 male or female body figures were presented to individuals to assess their perceptions of their own bodies (perceived body image, PBI) and ideal body (ideal body image, IBI) (in both cases, 1 = excessively obese, 8 = excessively thin and flaccid, and 15 = excessively muscular). In addition, body dissatisfaction was assessed by a single face-valid item (“How satisfied are you with your current body weight and appearance?” Response options ranged from 1 = extremely dissatisfied to 7 = extremely satisfied) [16]. Body satisfaction is considered as a key dimension in the evaluative–subjective component of body image [60].

The stigma associated with overweight and obesity was assessed with the Spanish version [61] of the 11-item Weight Bias Internalization Scale (M-WBIS) [62]. Weight-related stigma was assessed with respect to personal competence and self-worth, attractiveness, judgments by others, desire to lose weight, weight-related distress, sexual opportunities, and so forth (1 = completely disagree, 7 = completely agree). A global score was obtained by adding response values and then dividing by the number of items, with higher scores indicating greater self-stigma. The Cronbach’s α value was 0.83 in the present study.

Positive functioning was assessed with the Spanish version [63] of the 8-item Positivity Scale [64]. Positivity is defined as the tendency to view life and personal experiences from a constructive perspective, i.e., life satisfaction, personal confidence, self-pride, hope and enthusiasm for the future, social support, and so forth (1 = completely disagree, 5 = completely agree). A global score was obtained by adding response values and then dividing by the number of items, with higher scores indicating greater positivity. The Cronbach’s α value was 0.87 in the present study.

SWB was self-reported with the Happiness Scale [65]. Only the single-item indicator of current happiness (“How happy are you at present, i.e., the last few days or weeks?” Scores ranged from 0 = extremely unhappy to 10 = extremely happy) was used. Single-item indicators of happiness are usually used in national surveys and individual research [66]. Instead of specific indicators of satisfaction with life or hedonic balance (i.e., positive and negative affect) [67], we measured SWB at the molar level by assessing an individual’s summary assessment of his/her subjective happiness as a more global psychological phenomenon.

Adherence to a healthy diet was assessed with the 14-point Mediterranean Diet Adherence Screener (MEDAS, also known as PREDIMED) [68,69], which assesses adherence to the MedDiet with 14 dichotomic items for different diet nutrients (0 = no adherence to recommendations and 1 = adherence to recommendations). However, the MEDAS scores for some items (e.g., wine, meat), some absences (e.g., cereal and dairy products), and some other limitations (e.g., not taking into account culinary practices, average intakes, serving sizes, combinations of foods, and total energy intake) raise questions about the scoring decisions and cutoff points used for computing scores and the categories of adequate, moderate, and poor adherence. Moreover, the MEDAS results show low-to-medium correlations with other accepted tools for assessing adherence to the MedDiet [55,70,71]. These limitations are only in part addressed by a new, nonvalidated, 17-item version of the MEDAS [72]. Thus, since criticisms have been expressed regarding the classification of individuals based on their dietary habits as measured by their global score [30,73], we decided to use a single-nutrient approach and considered one of the most relevant or common indicators of adherence to a healthy diet, namely, the daily consumption of FV, foods at the base of the MedDiet pyramid [74]. Higher intakes of FV have consistently been identified as characteristics of healthy eating patterns both in the MedDiet [75] and other healthy diets [76] and a non-linear threshold effect of 800 g per day (i.e., about 5 servings a day) has been established for health endpoints [77]. Thus, we used the two items from the MEDAS collecting data on FV intake (items 3 and 4): “Consumption of ≥2 servings (200 g) of vegetables per day (at least 1 in salad or fresh; garnish or accompaniments, 100 g)” and “Consumption of ≥3 fruit units per day (including fruit juices)”. Then, based on the participants’ responses (yes = 1/no = 0), we classified participants into two profiles: healthy eaters were those reporting the consumption of at least 5 units/serving of both fruits and vegetables per day, whereas unhealthier eaters were those not consuming at least 5 units/serving of vegetables and fruits per day. A total of 54% of participants were classified as healthy eaters, while 46% were classified as unhealthy eaters.

### 2.3. Statistical Analyses

This research was a cross-sectional correlational study. The nature and adequacy of the data were checked, and parametric assumptions were confirmed before conducting analyses. Descriptive and other inferential results are reported elsewhere [13,17]. For the present study, a two-step cluster analysis was conducted, combining hierarchical agglomerative preclustering and then, for replication, a nonhierarchical iterative k-means cluster analysis for fixed solutions from two to four clusters, to identify psychosocial and diet-related profiles maximizing intraconglomerate homogeneity and between-conglomerate heterogeneity (with Euclidean distance as the method for distance measure). In this analysis, body image dimensions (PBI, IBI, and body satisfaction), self-stigma, adherence to a healthy diet (FV intake), positivity, and happiness were included to form the intraindividual profiles based on a one-factor analysis of variance (ANOVA) with post hoc pairwise comparisons (Bonferroni’s or Games–Howell’s comparisons were calculated based on Levene’s *F* test for homogeneity of variance) for determining the final variables contributing to clusterization. The optimal number of clusters was determined by the Bayesian information criterion (BIC) and confirmed by means of the pseudo-F (PSF) criterion or variance ratio. In addition, Goodman and Kruskal’s λ value and the percentage of cases correctly classified were also considered. This analysis was complemented by a discriminant analysis to further validate the classification. To establish possible differences between groups, a final ANOVA and pairwise comparisons (with corrections due to the homogeneity of variance) was conducted to explore the most contributing factors. One-way ANOVA was then run to explore the possible differences among the clusters due to objective BMI (as a continuous variable). Z scores were calculated for all the variables, except the indicator of a healthy diet. The significance level for all analyses was set at *p <* 0.05. Statistical analyses for the current study were conducted using SPSS 25.0 (Statistical Package for the Social Sciences, BMI^®^, Chicago, IL, USA).

## 3. Results

In examining the possible intraindividual configurations of body perceptions, body satisfaction, self-stigma, healthy diet, positivity, and SWB indicators, a three-cluster solution was chosen because this was the solution with the highest percentage of participants correctly clustered in each group, it had greater parsimony and replicability, and could be more readily interpreted in a meaningful way than other solutions. An initial ANOVA revealed significant differences among clusters for all configuration variables, and consequently, all the variables were included for the final cluster formation. Thus, three definitive clusters were identified (Table 1), a result supported by the PSF and λ values (*p* < 0.01), which reached optimal values for the three-cluster solution. 

**Figure 1:**
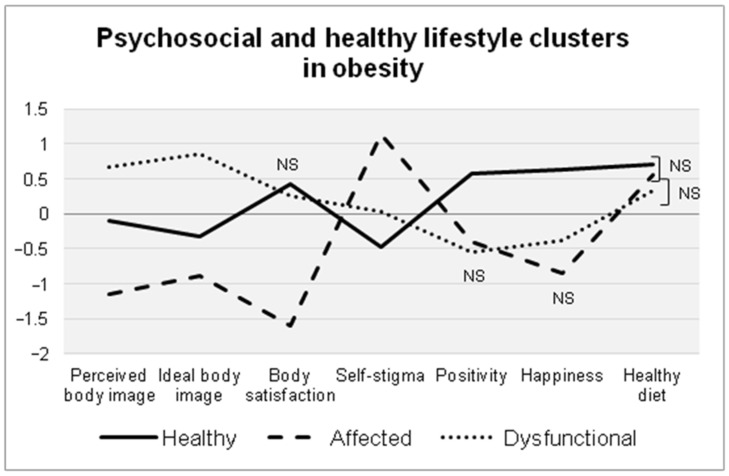
Graphical representation (centroids) of the psychosocial and healthy diet profiles identified in the cluster analysis. All the differences were significant at *p* < 0.05; NS = Non-significant difference (*p* > 0.05).

Specifically, significant differences were found between the clusters for all configuration variables (*p* < 0.05). However, Clusters 1 and 3 did not differ in terms of body satisfaction, and Clusters 2 and 3 did not differ in terms of positivity and happiness. Moreover, Cluster 2 did not differ from Clusters 1 and 3 in dietary habits, namely, FV intake (*p* > 0.05). Based on these profiles, we observed different configurations associated with different body appreciations, weight-related stigma, healthy lifestyle, positivity, and SWB (see Figure 1).

Each configuration was characterized by different psychosocial profiles: Cluster 1, composed of 46% of participants, was characterized by average perceptions of overweight and less exigent body ideals, which were slightly lower in weight and size compared to the participants’ current figures; individuals in this cluster reported the lowest weight-related self-stigma (−0.5 *SD*) and the highest levels of body satisfaction (≈0.5 *SD*), positivity (>0.5 *SD*), happiness (>0.5 *SD*), and adherence to a healthy diet (i.e., FV consumption) (0.7 *SD*). This cluster was therefore named the healthy group. 

Cluster 2, composed of 18% of participants, was characterized by showing higher perceived weight (>−1 *SD*) and more realistic body ideals (≈−1 *SD*), i.e., thinner yet closer to their actual weight; they reported the lowest levels of body satisfaction (>−1.5 *SD*) and happiness (≈−1 *SD*) and the highest self-stigma (>1 *SD*). In terms of their diet, this cluster reported some level of adherence to healthy eating (0.5 *SD*). This cluster was therefore referred to as the affected group. 

Cluster 3, composed of 36% of participants, was characterized by the most positive self-perceptions (0.7 *SD*) and the most exigent body ideals in terms of thinness and muscularity (≈1 *SD*); individuals in this cluster also reported a body satisfaction close to that of Cluster 1 but, at the same time, average self-stigma and, more importantly and resembling Cluster 2, lower happiness (≈−0.5 *SD*), the lowest positivity (−0.5 *SD*), and the poorest adherence to healthy eating (3 *SD*). Given this profile, this group was referred to as the dysfunctional group. 

Discriminant analysis for the overall model indicated high discriminant power (Wilks’ λ, *Χ^2^* = 84.252, *p* < 0.001), with 95% of the cases correctly classified. 

In addition, clusters were compared in terms of measured BMI (kg/m^2^). Significant differences were found (*F*_(2, 97)_ = 11.405, *p* < 0.001). While Clusters 1 and 3 showed similar BMIs (Cluster 1: *M* = 28.00, *SD* = 3.00; Cluster 3: *M* = 28.06, *SD* = 3.33; *p* > 0.05), Cluster 2 was composed of individuals with higher BMIs (*M* = 32.07, *SD* = 3.69) than Clusters 1 (*p* < 0.001) and 3 (*p* < 0.001).

## 4. Discussion

In the present study, we explored multidimensional profiles of experiences of excess weight, including perceptual and evaluative-subjective body image dimensions, self-stigma, healthy diet, positivity, and happiness in adults with overweight or obesity, as well as the association among these clusters with a validating variable, namely BMI. Three groups were identified based on these psychological and nutritional characteristics and BMI values were significantly different among these groups.

Individuals with obesity are usually classified according to BMI as they were homogeneous, but far from this supposed homogeneity, there is a large variability within the group, indicating that obesity management interventions must be tailored to individuals to be most effective [3]. Unfortunately, there has been relatively little consideration of the population-level heterogeneity of those individuals classified as obese; in addition, the extant research has focused on limited samples and risk or pathological factors. Since the psychosocial factors related to obesity are highly interrelated, it is inappropriate to study them independently from each other, and a profile-based approach combining various of such factors can be more adequate for understanding their overall impact. The present study contributes to the literature by exploring such configurations, including both risk and protective factors, in individuals with a wide range of BMIs in the categories of overweight and obesity. 

Our results support the existence of different profiles of individuals with excessive weight in terms of their psychosocial resources and lifestyle factors. The findings confirm the key role of body image dimensions, weight-related stigma, diet-related indicators, positivity, and happiness for overall well-being in individuals with overweight and obesity. Individuals in the largest subtype (46% of participants) were characterized as having more realistic body perceptions and body ideals, the highest level of body satisfaction, the lowest weight-related self-stigma, and the highest levels of positivity and happiness. These individuals also adhered to a healthy diet in terms of FV intake (at least compared to the cluster of most dysfunctional individuals). This cluster seems to support a healthy subgroup of individuals with excess weight who are conscientious of their weight, accept their body, take control of its management, and do not allow weight- and body-related pressures to make them feel unfortunate. We expected to find this more adaptive/high functioning subgroup based on the reviewed literature [1,2,3,4,10,11]. 

The smallest subgroup (18%) comprised affected individuals, indicating weight-related subjective distress, social disadvantage and, probably, being discriminated against due to obesity. This subtype was characterized by having higher perceived weight, the lowest level of body satisfaction, and less exigent body ideals (closer to their actual weight); they reported the highest self-stigma, low positivity, and the lowest level of happiness. This cluster summed up the two maladaptive profiles that we expected to find [1,2,3,4,10,11], one of which was expected to be more weight-based distressed and the other which was expected to be more concerned with the body. This profile indicates that these individuals, who seem to be aware of their weight and unhappiness, are intimately “suffering” for being overweight and obese. In terms of their diet, individuals in this cluster do not fully adhere to healthy eating for weight and appearance control, but it is possible that they try to do so by this means. This is something that should be addressed by future research. 

Unexpectedly, yet some previous findings pointed to it [2], there was a third profile (36%) containing dysfunctional individuals with apparently higher unawareness of their excessive weight and associated risks because they reported more positive—probably unrealistic, illusory—self-perceptions and high body satisfaction but, at the same time, more exigent body ideals. Nevertheless, these individuals also reported high self-stigma and low positivity and happiness as well as the poorest adherence to healthy eating. Contrary to the abovementioned maladaptive, internally focused subgroup, these individuals seem to have a more social, external focus regarding weight and appearance based on sociocultural beauty standards and social pressures concerning their body and weight. Tentatively, this profile could perhaps be attributed to weight preoccupation and body dissatisfaction related to more external factors (e.g., social pressure to be thin, desire for social acceptance), instead of internal feelings of eating control or negative mood, something that future research should explore. Moreover, it would be of interest to explore the risk for eating disorders and general psychopathology in both maladaptive subgroups, in future research. 

Our findings support previous profiles of individuals with excessive weight with similar clusterization variables, such as body- and weight-related concerns [1,10], eating practices [2,3,4,11], and SWB-related variables [2,3,10]. Both females and males with obesity experience their excessive weight in several ways, with different degrees of shape and weight worries, negative self-evaluations, and body dissatisfaction, probably derived from thinness-oriented beauty ideals, anti-fat social pressures, and weight-related stigma [10]; of adherence to nutritional recommendations [4]; and of psychological well-being [3]. None of these studies examined the influences of weight-related stigma or personality resources, and research on positive states of well-being is lacking. Whereas body and eating concerns, eating disturbed behaviours, personality and temperament traits, and overall psychopathology have been used as external variables [2,9,11], the present research incorporated a wide range of psychosocial and lifestyle factors as configuring variables. By incorporating such variables, our findings supported the existence of an adaptive and a maladaptive profile claimed in studies such as those by Jansen et al. [10] and Perdue et al. [1]. As Caroleo et al. [11] found, a subgroup of individuals with excessive weight emerged who seem to be obese due to overeating and do not “suffer” for being obese, but are aware of their weight and try to manage it (healthy subgroup), paralleling the cluster of individuals with hyperphagia and no evidence of relevant psychopathology of their study. Our findings also support the existence of a subgroup of individuals who show higher weight-related distress, paralleling Caroleo’s et al. subgroup of individuals with increased body image concerns, more altered eating behaviours, and higher levels of psychopathology, who, in our study, were further subclassified into a subgroup of highly distressed individuals, with abnormal psychological features (affected subgroup), and another subgroup of abnormal body- and eating-related features (dysfunctional subgroup). Moreover, our results parallel, in part, the findings of Gagnon-Girouard et al. [2], with the healthy profile mirroring their subgroup with a more adaptive profile, and the dysfunctional and distressed profiles mirroring their restrained and depressive subgroups, respectively, which showed progressively more impaired profiles in terms of body, eating, mood, and overall psychopathology.

With respect to BMI, it has been generally used as an external variable for validation. Previous empirically based classifications have not supported that profiles of obesity differ according to weight-related factors [1,2,4,9,10]. This finding tends to support that typologies appear to be more closely associated with multidimensional psychosocial functioning than with the overweight level per se. Such research has, however, been conducted with samples limited to high-risk populations and bariatric candidates/patients, mainly high-level obesity and female participants, which can be seen as more homogeneous compared to community-based samples comprising a wide range of BMIs, such as the sample in the present study. When such wider samples are used, contrary findings are obtained. In support of previous findings pointing out that subtypes of individuals with worse psychosocial features also have higher BMIs [3,11], we found differences across the three subgroups, also indicating that those with the highest obesity level reported the poorest psychosocial profile.

Our findings have theoretical and clinical utility, guiding both research and treatment. The clustering of individuals into different profiles and the identification of the underlying psychological mechanisms for each distinct subgroup of individuals with overweight and obesity would foster more suited, individualized counseling and a more effective care approach. One advantage of the present work is a novel approach integrating protective factors into the configuration of psychosocial profiles of individuals with excess weight. Thus, the present study is in accordance with a shift from a weight-centered paradigm for body valuation and obesity management with BMI oriented targets to an alternative paradigm focused on patients’ self-acceptance, decreased body-related worry, enhancement of healthy habits (eating patterns, physical activity), and the pursuit of health and well-being [13]. Interventions for overweight and obese individuals should include addressing body-related perceptions and weight and shape concerns with actions focused on inclusive, positive aesthetic models. An effort for combating the ubiquitous social weight stigmatization and mitigating the deleterious influence of self-stigma should also be promoted. Moreover, instead of only focusing on restrained and eating disorder symptoms [2,11], a focus on adherence to healthy diet recommendations can be adopted. We thus encourage the promotion of healthier, positive, and realistic body images [78] and of healthy weight-control strategies for weight and appearance management [79]. In addition, although previous evidence has suggested that happiness is lower among individuals with obesity, research on this issue is warranted to increase our knowledge. Thus, our findings are expected to make a relevant contribution to the knowledge on the protective factors for overall well-being in obesity. In sum, interventions should not target obese individuals as a whole, but tailor strategies depending upon the subgroup to which individuals belong. Future research exploring the predictive validity of such subtyping-based interventions in terms of treatment type, prognostic course, outcome, weight loss success, personal satisfaction, and general well-being within each cluster is thus needed.

Despite the contributions of the present study, which included the consideration of several dimensions of body perception, weight stigma, dietary habits, positivity, and happiness in men and women with overweight and obesity, our conclusions should be interpreted in light of some limitations. First, the main limitation is the preference for cluster analysis, as it is a descriptive analysis and the solution is not unique, but depends upon the analyst’s choices and other conditions, such as the participants’ characteristics, the included variables, the selected number of clusters, and the decided distance metric. 

Second, the sample was limited in size and constituted a nonrandom sample of individuals with excessive weight who, in general terms, revealed an underestimation of their real weight and desired a slightly slimmer body, who reported, on average, moderate body satisfaction, low self-stigma, high positivity, and high happiness, and who were interested in their health and in managing or losing weight. We do not know whether the obtained clusters are unique to people with these conditions, and thus, not generalizable to other obese individuals. Thus, our findings need to be replicated with broader and more heterogeneous samples, including more individuals with obesity types II and III. In this regard, the characteristics and size of the sample might have made it impossible to detect more possible clusters or more heterogeneous groups, with other configurations being masked by this conformation of the sample. 

Third, our study relied on self-report measures that may be susceptible to various errors and biases. Further research using multimodal assessment is warranted. Moreover, other psychosocial variables were ignored in configurating the clusters (e.g., mental and physical health indicators, quality of life, stress, social support, eating pathology). Future research should include a wide range of personality, cognitive, motivational, emotional, behavioural, and psychosocial factors. Besides, since contradictory findings have been obtained for age [2,9,11 vs. 3,4,10] and sex-gender [9 vs. 11], future research should explore the contribution of such variables. Previous findings with the same sample [13] have revealed sex–gender-based differences in terms of body perceptions and weight-related self-stigma, probably due to the higher sociocultural relevance imposed on the feminine body and females’ higher internalization of beauty standards compared to males [60]. Further analyses revealed no other influences of age or sex–gender, e.g., in terms of positivity and SWB. Nevertheless, differences in all the variables, except positivity, were found between individuals with obesity and with overweight. Thus, we decided to explore only BMI as an external criterion variable. In addition, only one previous study has explored socioeconomic factors [10]. Due to the socioeconomic characteristics of our sample, we discarded that analysis. As a consequence, future research needs to include a broader range of variables to validate individual subtypes and more validating, external variables. 

In addition, obesity is contextually constructed, both in terms of understandings and causations. Scholars and experts are stressing the increasing explanatory power of sociocultural factors that generate obesogenic environments, beyond biological and behavioural factors [80,81]. Thus, beliefs and attitudes towards excessive weight held by non-obese people, obese people, children and adolescents, educators, employers, healthcare professionals, and policy makers; health-related choices and lifestyle practices; relationships with individuals with excessive weight in all spheres of daily life; and, broadly, social weight-centered discourses on obesity, are influenced by a myriad of sociocultural factors in intersection with other influences coming from ethnic, socioeconomic, age, gender, literacy, employment, rural/urban living, and migration factors, among others. These interactions have a role on several endpoints, including local prevalence rates, clinical management, stigmatization, and personal and social lived experiences with obesity. Sociocultural influences start in early childhood [82,83]. For instance, influences on lifestyle of food-related advertising [84] and consumption–leisure activities (e.g., abusive use of videogames or online activities) [85] in children and adolescents are increasingly being explored. This translates in the necessity of tailoring interventions more specific to target populations, dealing with obesity in a holistic and cross-disciplinary manner to fully understand all of its dimensions and to successfully give answer to the needs of individuals with excess weight from a startpoint of respect and help, beyond exclusively biomedical solutions that lead to medicalization, blaming and shaming, and discrimination of persons based on body size, appearance, or behaviour [86,87]. By focusing in a Mediterranean nation as Spain, we could not address the complete social and cultural background of obesity as a global social problem.

Finally, due to the descriptive, correlational, and cross-sectional nature of the data, causal inferences could not be stated, and the utility of our solution may best be determined through future longitudinal and experimental analyses (e.g., whether cluster membership relates to intervention efficacy).

## 5. Conclusions

Despite these limitations, our results are pioneer and interesting. In summary, this investigation provides initial evidence that three psychosocial and eating-based groups can be identified in adults with overweight and obesity that differ in their configurations of body image dimensions, weight-related stigma, eating habits, positivity, and well-being (i.e., happiness). In support of the uniqueness of these groups, the profiles were validated with BMI as an external variable. Thus, a cluster of healthier individuals with excessive weight (≈1/2 of the sample) was identified, who hold more realistic self-perceptions, better psychosocial features, and healthier diet in terms of FV consumption; a cluster of affected individuals (≈1/5 of the sample), who hold the most negative self-perceptions, the poorest psychosocial profile, and the highest BMI; and a cluster of dysfunctional individuals (≈1/3 of the sample), who seem to hold unrealistic body self-perceptions accompanied by poor psychosocial features, and comparatively the poorest adherence to healthy eating. Our findings highlight the relevance of addressing these parameters in the management of obesity due to the heterogeneity of individuals in such population. The current study also offers new directions for the study of well-being in obesity. Future research should investigate the ways in which weight, body satisfaction, weight-related stigma, dietary patterns, positivity, and happiness may interact to affect functioning and well-being in individuals with excess weight and to explore how accumulated evidence may be used to inform health interventions for preventing and managing obesity in all its dimensions. The identified clusters may be used to tailor interventions to enhance their effectiveness. Studies exploring the results of different types of treatments according to these profiles may be useful for testing the clinical validity of the behavioural and psychosocial distinctions among individuals with obesity.

## Figures and Tables

**Table 1:** Means (centroids), standard deviations, and between-group comparisons (final ANOVA) for three clusters (Z scores).

Configurational Variables	Healthy N = 46%		Affected N = 18%		Dysfunctional N = 36%		*F*	*p*
	*M*	*SD*	*M*	*SD*	*M*	*SD*		
Perceived body image	−0.09	0.62	−1.14	0.48	0.68	1.04	33.092	0.000 **
Ideal body image	−0.33	0.83	−0.88	0.64	0.86	0.66	41.895	0.000 **
Body satisfaction	0.42	0.58	−1.59	0.82	0.25	0.69	62.701	0.000 **
Self-stigma	−0.48	0.62	1.15	1.00	0.04	0.93	25.637	0.000 **
Positivity	0.58	0.65	−0.40	1.10	−0.54	0.93	20.046	0.000 **
Happiness	0.63	0.62	−0.84	1.07	−0.38	0.86	27.933	0.000 **
Healthy diet	0.70	0.47	0.56	0.51	0.33	0.48	5.808	0.004 **

** *p* < 0.01.

## Data Availability

Data available on request due to restrictions. The data presented in this study are available on request from the corresponding author. The data are not publicly available due to privacy.
